# Androgen Action via Testicular Arteriole Smooth Muscle Cells Is Important for Leydig Cell Function, Vasomotion and Testicular Fluid Dynamics

**DOI:** 10.1371/journal.pone.0013632

**Published:** 2010-10-26

**Authors:** Michelle Welsh, Richard M. Sharpe, Lindsey Moffat, Nina Atanassova, Philippa T. K. Saunders, Sigrid Kilter, Anders Bergh, Lee B. Smith

**Affiliations:** 1 Medical Research Council Human Reproductive Sciences Unit, Centre for Reproductive Biology, The Queen's Medical Research Institute, Edinburgh, United Kingdom; 2 Institute of Experimental Morphology and Anthropology with Museum, Bulgarian Academy of Sciences, Sofia, Bulgaria; 3 Department of Anatomy and Pathology, University of Umeå, Umeå, Sweden; University of Córdoba, Spain

## Abstract

Regulation of blood flow through the testicular microvasculature by vasomotion is thought to be important for normal testis function as it regulates interstitial fluid (IF) dynamics which is an important intra-testicular transport medium. Androgens control vasomotion, but how they exert these effects remains unclear. One possibility is by signalling via androgen receptors (AR) expressed in testicular arteriole smooth muscle cells. To investigate this and determine the overall importance of this mechanism in testis function, we generated a blood vessel smooth muscle cell-specific AR knockout mouse (SMARKO). Gross reproductive development was normal in SMARKO mice but testis weight was reduced in adulthood compared to control littermates; this reduction was not due to any changes in germ cell volume or to deficits in testosterone, LH or FSH concentrations and did not cause infertility. However, seminiferous tubule lumen volume was reduced in adult SMARKO males while interstitial volume was increased, perhaps indicating altered fluid dynamics; this was associated with compensated Leydig cell failure. Vasomotion was impaired in adult SMARKO males, though overall testis blood flow was normal and there was an increase in the overall blood vessel volume per testis in adult SMARKOs. In conclusion, these results indicate that ablating arteriole smooth muscle AR does not grossly alter spermatogenesis or affect male fertility but does subtly impair Leydig cell function and testicular fluid exchange, possibly by locally regulating microvascular blood flow within the testis.

## Introduction

Testosterone is essential for normal male fertility, controlling development of the male reproductive system [1] and the later initiation and maintenance of spermatogenesis [2], [3]. Testosterone is produced by the testicular Leydig cells (LC) and binds to the androgen receptor (AR) to modulate gene transcription in target cells [4]. ARs are expressed in several testicular cell types [5], [6] and recent investigations showed that ablating AR specifically from Sertoli cells (SC) or peritubular myoid cells (PTM) results in male infertility [7], [8], [9]. Conversely, germ cells (GC) lacking AR develop normally [10], [11]. AR is also expressed in other testicular cell types including the arteriole smooth muscle cells [6], [12], and it has been suggested that androgen signalling via the arterioles may play a role in regulating testicular function and male fertility [12]; however, how androgens mediate these effects remains unknown.

The vascular system forms early in testis development [13], [14] and normal testis function depends on the vascular system delivering oxygen, nutrients and hormones into testicular interstitial fluid (IF) and removing waste and secretory products; for example testicular blood flow positively correlates with serum testosterone concentrations [15]. There are several aspects of blood flow in the testis that are unique, including low blood oxygen tension and non-pulsatile blood flow [16], [17]. Furthermore, the bulk of the testis (i.e. the highly proliferative seminiferous tubules) is avascular therefore it relies on IF for transport of nutrients and hormones; this fluid transport itself is influenced by hormones, for example luteinising hormone (LH) increases testicular vascular permeability [17] by increasing vascular endothelial growth factor (VEGF) production [12], [18], [19]. Maintaining normal blood flow through the testicular microvasculature relies on a balance of events at the pre- and post-capillary beds and it has been suggested that steroid hormones may play a role in regulating this [12], but the exact mechanisms remain under explored.

Testicular microvasculature blood flow is thought to be regulated locally and shows regular rhythmical variations, known as vasomotion [20], [21], [22]. Vasomotion is present in most vascular beds [17], is independent of heart beat, can be influenced by hypoxia and hormones [20], [21], [22], [23], [24], and is thought to be mediated by the vascular smooth muscle cells [17]. Vasomotion mediates the transfer of fluids between blood plasma and IF, therefore fluid transfer, and hence IF volume and content, is easily influenced by changes in testicular blood flow [25] and vasomotion [20], [21], [22]. When the testicular microvasculature dilates, blood flows through quickly and plasma filters from the blood into the interstitium, whilst slow or stops in flow due to microvasculature constriction allows fluids to filter from the interstitium back into the blood [22]. Several lines of evidence point to testosterone as one of the key factors controlling vasomotion and testicular blood flow, which in turn affects fluid dynamics and testis function [17], [26].

In the testis, ARs are expressed in arteriole smooth muscle cells, but not in arteriole endothelial cells or in venules or capillaries [6]. Interestingly, ARs are not expressed in the arteriole smooth muscle cells in all organs (e.g. the kidney [6]), therefore suggesting a specific local role for AR in the testicular vasculature. AR expression in testicular arterioles depends on sufficient intra-testicular testosterone concentrations and signalling via these ARs may mediate some of the effects of androgens on testicular microcirculation [6]. Alternatively, testosterone could affect blood flow by signalling via the LCs or other AR positive testicular somatic cells, such as the SC or PTM cells, which in turn signal to the blood vessels. The aim of our studies was to directly investigate the role for testicular vascular smooth muscle AR in testis function and male fertility.

## Materials and Methods

### Breeding of transgenic mice

We generated mice in which the AR has been selectively ablated from the arteriole smooth muscle cells using Cre/*loxP* technology; some of the mice used in this study were generated previously in a comparative study of the specificity of the sm22-Cre line and the MHC-cre line [8]. Male mice heterozygous for Cre Recombinase under the control of a smooth muscle 22 α [27] promoter were mated to female mice homozygous for a floxed AR [7]; all mice are maintained on a C57BL6 background. As previously reported [8], *Cre* positive (AR^flox^ positive) male offspring from these matings are termed SMARKO, while the Cre negative AR^flox^ positive littermates were used as controls (termed control or C). *Cre* positive, AR^flox^ negative mice were also generated to confirm that expression of *Cre* alone did not induce a phenotype [8]. All mice were bred under standard conditions of care and use under licensed approval from the UK Home Office (Project licence 60/3544). Mice were genotyped from ear or tail DNA for the presence of *Cre* and/or AR^flox^ using standard PCR ([8] and http://jaxmice.jax.org/protocolsdb/f?p=116:2:3392087113007235::NO:2:P2 _MASTER_PROTOCOL_ID,P2_JRS_CODE:288,004746); the internal positive control used in the Cre genotyping assay is Interleukin-2, as recommended by the Jackson Laboratory.

### Evaluation of fertility

To investigate fertility, d100 SMARKO and control males were each housed with an adult C57BL/6J female for 4 days. This was repeated with 3 subsequent C57BL/6J females per male. Female mice were monitored for 25 days for litters to be born.

### Recovery of reproductive tissues

Male mice were culled at various postnatal ages (d12–d300) and reproductive tissues were collected, weighed and snap-frozen or fixed in Bouins as previously described [8]. Bouins fixed tissues were processed and embedded in paraffin wax and 5 µm sections were cut for histological analysis as previously described [28]. Sections of testis and epididymis were stained with hematoxylin and eosin using standard protocols and examined for histological abnormalities.

### Hormone analysis

Immediately after culling, blood was collected from d50 and d100 KO and control mice by cardiac puncture and sera were assayed for FSH, LH and testosterone as previously published [29], [30]. Intra-testicular testosterone concentrations were measured as previously published [31]. All samples were run in a single assay for each hormone, and the within-assay coefficients of variation were all <10%.

### Treatment with hCG

SMARKO (n = 6) and control (n = 15) adult male mice were injected subcutaneously with 20IU hCG in 0.2 ml saline (Organon Laboratories, Cambridge, UK) 16 h prior to culling. Mice were culled and blood was collected by cardiac puncture to measure serum testosterone and testes were recovered and weighed, as detailed above. IF was collected from testes as previously published [32], [33] and assayed for testosterone.

### Vasomotion measurements

Vasomotion was measured in SMARKO (n = 7) and control (n = 13) d100 male mice using previously published Doppler flowmeter methodology [21], [22]. Mice were anaesthetised by subcutaneous injection of 10 ml/kg ketaminol (10 mg/ml) and narcoxyl (1 mg/ml) in sterile water. The probes, which measure flow in a tissue volume of approximately 2 mm^3^, were held with a micromanipulator approximately 1 mm above an area of the testicular surface devoid of large vessels. The laser Doppler flow signals were recorded for at least 5 min on the surface of both testes and average blood flow and vasomotion frequency were later analysed using the software Perisoft for Windows 1.01 (Perimed AB). As focal areas of seminiferous tubule abnormalities could be identified in SMARKO testes, flow was examined at multiple spots over each testis surface in both control and KO mice to ensure that data represented vasomotion throughout the testis and not just in ‘normal’ or ‘abnormal’ areas; no obvious differences were seen in recordings at different locations within each tests.

### Determination of testicular cell composition

Standard stereological techniques involving point counting of cell nuclei were used as previously described [7], [34], to determine the nuclear volume per testis of SC, GCs, LCs, blood vessels and the relative volumes of interstitium, and seminiferous tubule lumen in SMARKO and control mice at d100 (n = 4–6). In brief, testicular cross sections were examined using a Leitz 363 plan apo objective lens (x63) fitted to a Leitz Laborlux microscope and a 121-point eyepiece graticule (Leica Microsystems, Wetzlar, Germany). For each animal, 32–64 microscopic fields were counted, and values for percent nuclear volume were converted to absolute nuclear volumes per testis by reference to testis volume ( =  weight). SC and LC nuclear size, blood vessel number and size, proportion interstitium and lumen, and seminiferous tubule diameter were determined using an Olympus Optical BH-2 microscope (Olympus Optical Co., Tokyo, Japan) fitted with a Prior automatic stage (Prior Scientific Instruments, Cambridge, UK) and Image-Pro Plus (version 4.5.1) with Stereologer-Pro 5 plug-in software (Media Cybernetics, Bethesda, MD, USA). The ‘abnormal’ seminiferous tubules were not excluded from these analyses. Data were used to determine: (*i*) the relative contribution of seminiferous tubule lumen and interstitium to the testis volume (expressed as a % and as actual volume per testis); (*ii*) average seminiferous tubule diameter; (*iii*) the nuclear volumes of and number of SCs and LCs per testis; (*iv*) the nuclear volume per testis of each type of GC; and (v) the average number and size of blood vessels per testis.

### Immunohistochemical analysis

As well as PCR genotyping, d100 mice were examined by immunohistochemistry for the presence of *Cre* using a previously published method [8]. Single colorimetric immunohistochemistry was also performed on d100 testes for WT-1 to demonstrate the location of SC nuclei and for 3β-HSD to highlight LCs [8]. Briefly, sections were deparaffinized, rehydrated, and antigen retrieved before blocking of nonspecific binding sites, as detailed previously [28]. Sections were incubated overnight at 4°C with the primary antibody diluted accordingly as detailed in Table 1. Immunostaining was detected using the secondary antibody and detection system specified in Table 1. Diaminobenzidine (DAB) immunostained slides were counterstained with hematoxylin, dehydrated, and mounted with Pertex (Histolab, Gothenburg, Sweden), and images were captured using a Provis microscope (Olympus) equipped with a Kodak DCS330 camera (Eastman Kodak, Rochester, NY, USA). Fluorescent immunostained sections were mounted in Mowiol mounting medium (Calbiochem, San Diego, CA, USA), and fluorescent images were captured using a Zeiss LSM 510 Meta Axiovert 100 M confocal microscope (Carl Zeiss Ltd., Welwyn, UK). To ensure reproducibility of results representative testes from at least 3 animals at each age were used, and sections from SMARKO and control littermates were processed in parallel on the same slide on at least 2 occasions. Slides were incubated with normal serum alone as a negative control to ensure that any staining observed was specific.

**Table 1:** Immunohistochemistry antibody details.

Antibody		Antibody source	Dilution	Detection system
3βHSD		Santa Cruz (Santa Cruz, USA)	1∶4000	Tyramide 546
AR		Santa Cruz	1∶50	Goat anti-rabbit alexa 488
Cre/SMA	Cre	Abcam	1∶5000	Tyramide 488
	SMA	Sigma	1∶500	Goat anti-mouse alexa 633
WT-1		Dako	1∶1000	Goat anti-mouse biotinylated + DAB

### RNA extraction and reverse transcription

RNA was isolated from d100 frozen testes from SMARKO or control mice, using RNeasy Mini extraction kit with RNase-free DNase on the column digestion kit (Qiagen, Crawley, UK) according to the manufacturer's instructions, and quantified using previously published methods [8]. Random hexamer primed cDNA was prepared using the Applied Biosystems TaqMan® reverse transcription kit (Applied Biosystems, Foster City, USA) according to manufacturers' instructions.

### Determination of deletion of AR exon 2

RT-PCR was performed on cDNA synthesised from testes from d100 SMARKO mice and control littermates using primers for *AR* exon 1 and 3, as previously described [8].

### Quantitative analysis of gene expression

Quantitative PCR was performed on d21 or 100 SMARKO and control testes for steroidogenesis enzymes and the LC differentiation marker Insulin-like factor 3 (Insl3). Primers are detailed in Table 2, using an ABI Prism 7500 Sequence Detection System (Applied Biosystems) and the Roche Universal Probe library (Roche, Welwyn, UK), as previously described [8]. The expression of each gene was related to luciferase, an external positive control, and all genes were expressed per testis.

**Table 2:** Taqman primer details.

Gene	Forward primer	Reverse primer
Steroidogenic acute regulatory protein (StAR)	ttgggcatactcaacaacca	Acttcgtccccgttctcc
3β-hydroxysteroid dehydrogenase (3βHSD)	tgtgaccatttcctacattctga	ccagtgattgataaaccttatgtcc
17β-hydroxysteroid dehydrogenase (17β HSD)	aatatgtcacgatcggagctg	gaagggatccggttcagaat
Cytochrome p450 11a (cyp11a or p450scc)	aagtatggccccatttacagg	tggggtccacgatgtaaact
Cytochrome p450 17 (cyp17 or 17aOH)	catcccacacaaggctaaca	cagtgcccagagattgatga
Vascular endothelial growth factor A (VEGF-A)	gtacctccaccatgccaagt	tgggacttctgctctccttc
Insulin-like factor 3 (Insl3)	aagaagccccatcatgacct	tttatttagactttttgggacacagg

### Statistical Analysis

Data were analyzed using GraphPad Prism version 5 (Graph Pad Software Inc., San Diego, USA) using a 2-tailed unpaired *t* test or a 1-way ANOVA followed by Bonferroni post-hoc tests. Values are expressed as mean ± SEM. Normality was confirmed using D'Agostino & Pearson omnibus normality test.

## Results

### Generation and characterisation of mice with a selective deletion of AR from blood vessel smooth muscle cells

**Figure 1:**
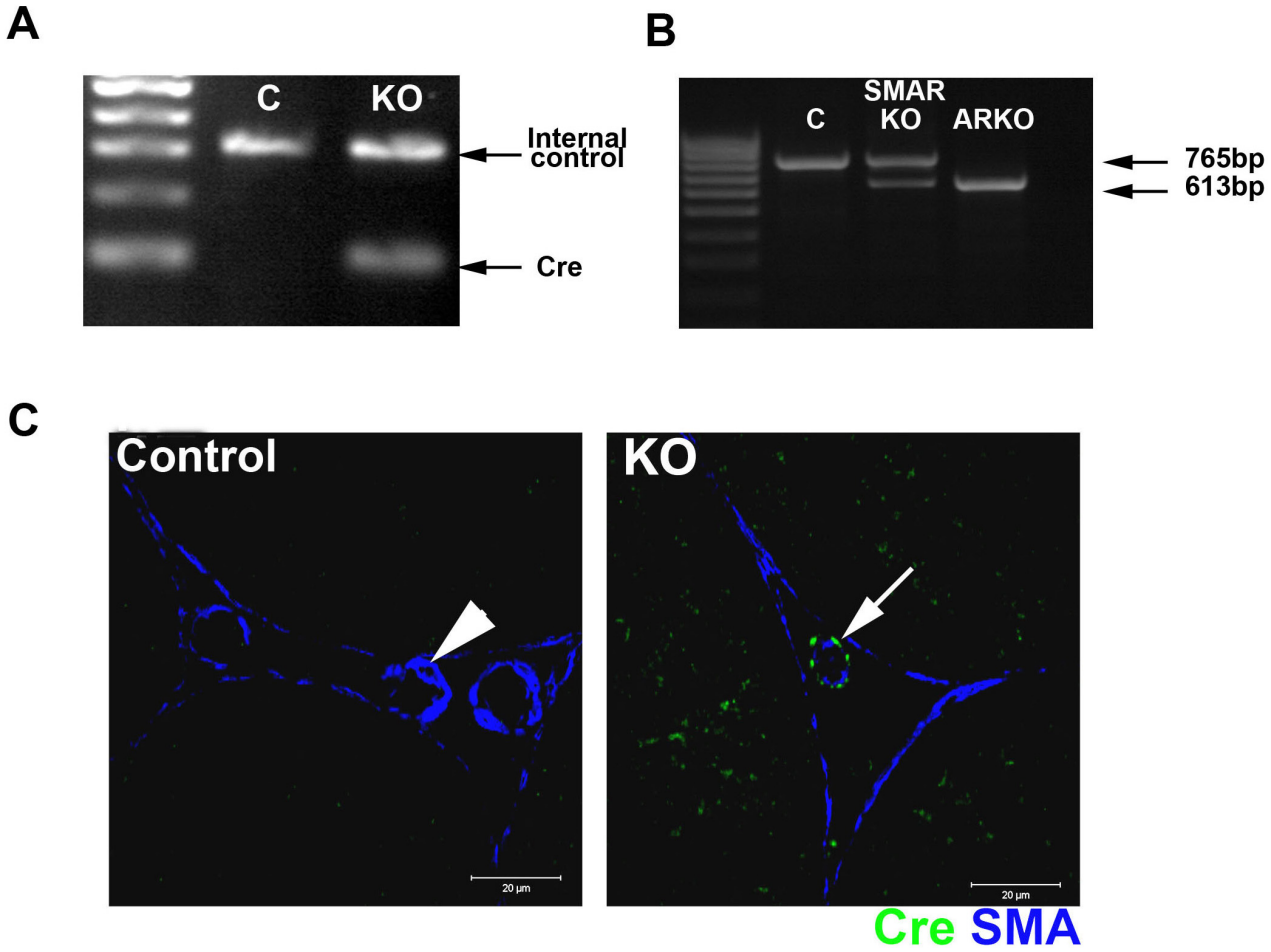
Characterisation of Cre Recombinase expression in adult SMARKO testes. **A.** Approximately 50% of male SMAR were *Cre* positive (KO), identified by the presence of a band at 100 bp. Control (C) littermates were negative for *Cre*. **B.** Deletion of androgen receptor (*AR*) in the testes was determined using RT-PCR spanning exon 2. Only the larger 765 bp wild-type (WT) AR band was seen in control testes while the smaller 613 bp KO band was seen in ARKO testes. Both bands were identified in SMARKO testes, showing deletion of AR in a proportion of cells. **C.** Immunohistochemistry for Cre Recombinase (green) and smooth muscle actin (SMA, blue) showed that Cre Recombinase was expressed selectively (arrow) in the smooth muscle cells surrounding the blood vessels in adult SMARKO testes but not in any other testicular cell types or in any cells in control testes (arrowhead).

As previously reported [8], no reproductive phenotype was identified in adult SM *Cre* positive, AR^flox^ negative male mice demonstrating that expression of *Cre* alone had no effect (data not shown). All SMARKO males were hemizygous for X-linked *AR^flox^* while approximately 50% of these males also carried the *Cre* transgene (representative example of genotyping results shown in Figure 1A; knockout - KO); the *AR^flox^* positive *Cre* negative male littermates were used as controls. To confirm that Cre Recombinase was functioning in these mice we assayed for the deletion of exon 2 of the *AR* in SMARKO testes by RT-PCR. As previously reported [8], SMARKO control testes only expressed the full length (WT) band whereas ARKO testes only expressed the smaller KO band, confirming that exon 2 of the *AR* had been deleted from all cells (Figure 1B); testes from SMARKO mice expressed both the WT and the KO band, demonstrating that *AR* had been deleted only from a proportion of testicular cells (Figure 1B). Immunohistochemistry was therefore undertaken in d100 testes to identify which cells in the testes expressed Cre Recombinase. As previously published [8], testes from Cre negative AR^flox^ positive control mice showed no specific testicular Cre Recombinase immunoexpression at d100, while every smooth muscle cell around the blood vessels immunostained for Cre Recombinase in SMARKO mice (Figure 1C). Cre Recombinase was never expressed in any other testicular cell type in SMARKO mice, including the smooth muscle peritubular myoid cells. The sm22-Cre mice were also used in a previous study to reportedly target AR in the peritubular myoid cells [35]; however, we [8] and others [36] have unequivocally demonstrated that the sm22 promoter drives Cre expression in the testicular arteriole smooth muscle cells but not in any other testicular cell type. Cre recombinase is also expressed in the arteriole smooth muscle cells outside the testis, such as the aorta [36]. We cannot completely rule out any global effect on blood flow affecting the testis, however, testicular blood flow is believed to be independent of heartbeat and regulated locally [20], [21], [22] therefore any effect on the aorta is unlikely to have any significant effect on the testis.

### Characterisation of the reproductive system in SMARKO mice

**Figure 2:**
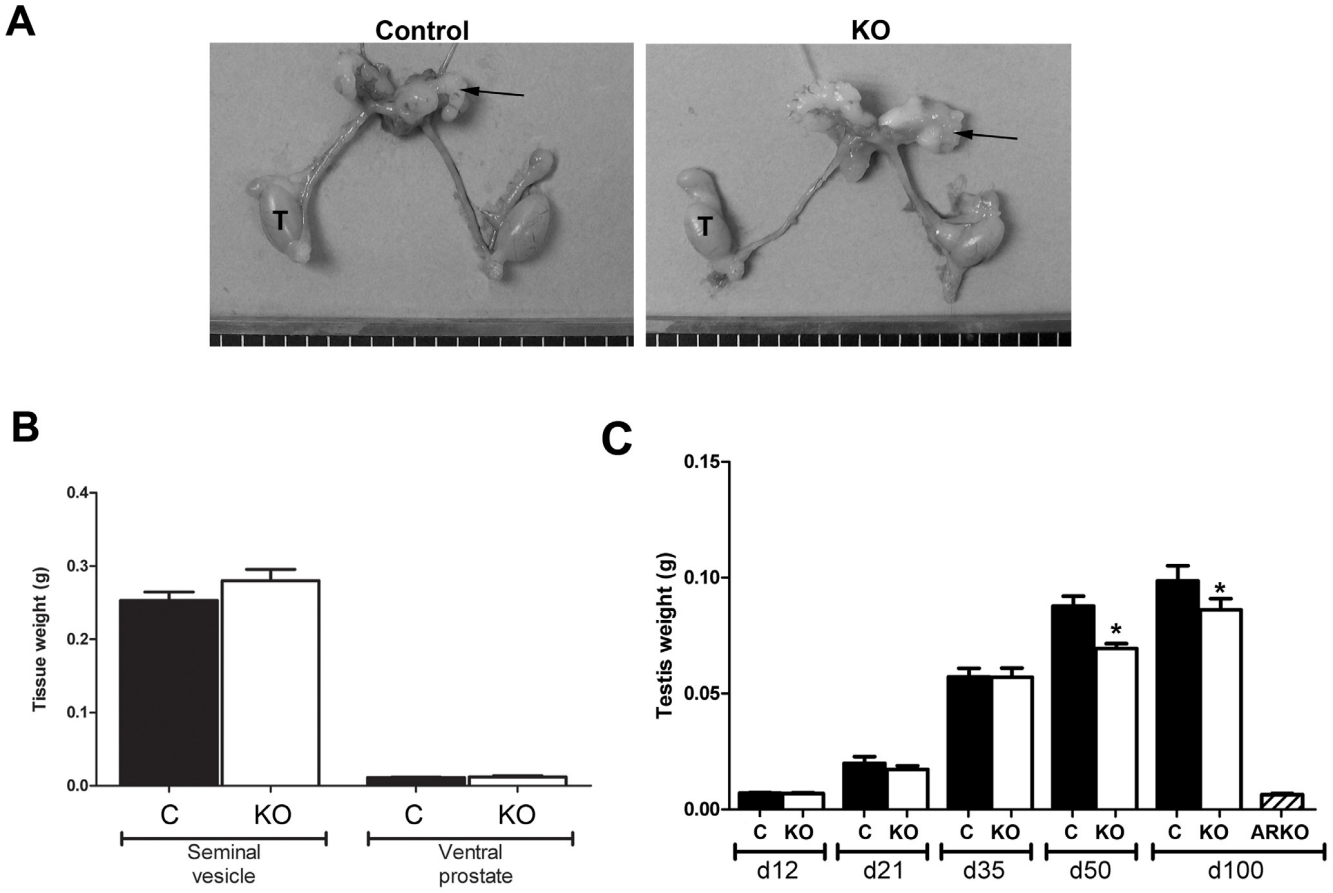
Gross morphology of the reproductive system from SMARKO mice. **A.** Urogenital tract from males at d100 in which the testes (T) are slightly smaller in the SMARKO than in controls while the seminal vesicles (arrow) are not different in size. **B.** Quantification of seminal vesicle and ventral prostate weight in SMARKO and control (C) mice at d100. **C.** Quantification of testis weight in SMARKO and control mice from d12-300. Values are means ± S.E.M. (n = 6–22 mice), * p<0.05 compared to controls.

Bodyweight was not significantly different between SMARKO and control males at any age (data not shown). SMARKO males were normally fertile (data not shown) and, in contrast to ARKO mice [7], the reproductive tract formed normally in SMARKO males (Figure 2A). Testis descent and external sexual development were normal in SMARKO males, with no significant reduction in anogenital distance (AGD) or penis length (data not shown). Seminal vesicle and ventral prostate weights were also normal in SMARKO males at d100, compared to control littermates (Figure 2B) and no obvious abnormalities were observed in the epididymis in SMARKO adult males (data not shown). Testis weight was normal at d12 -35 (Figure 2C) but was significantly smaller in SMARKO mice than in control littermates at d50 onwards (Figure 2C); note that testis weight did not change significantly in the KO males between d100 and d300. Expression of *Cre* alone, without AR^flox^ expression, had no effect on testis weight at d100 (data not shown).

### Postnatal testis morphology and cell numbers

**Figure 3:**
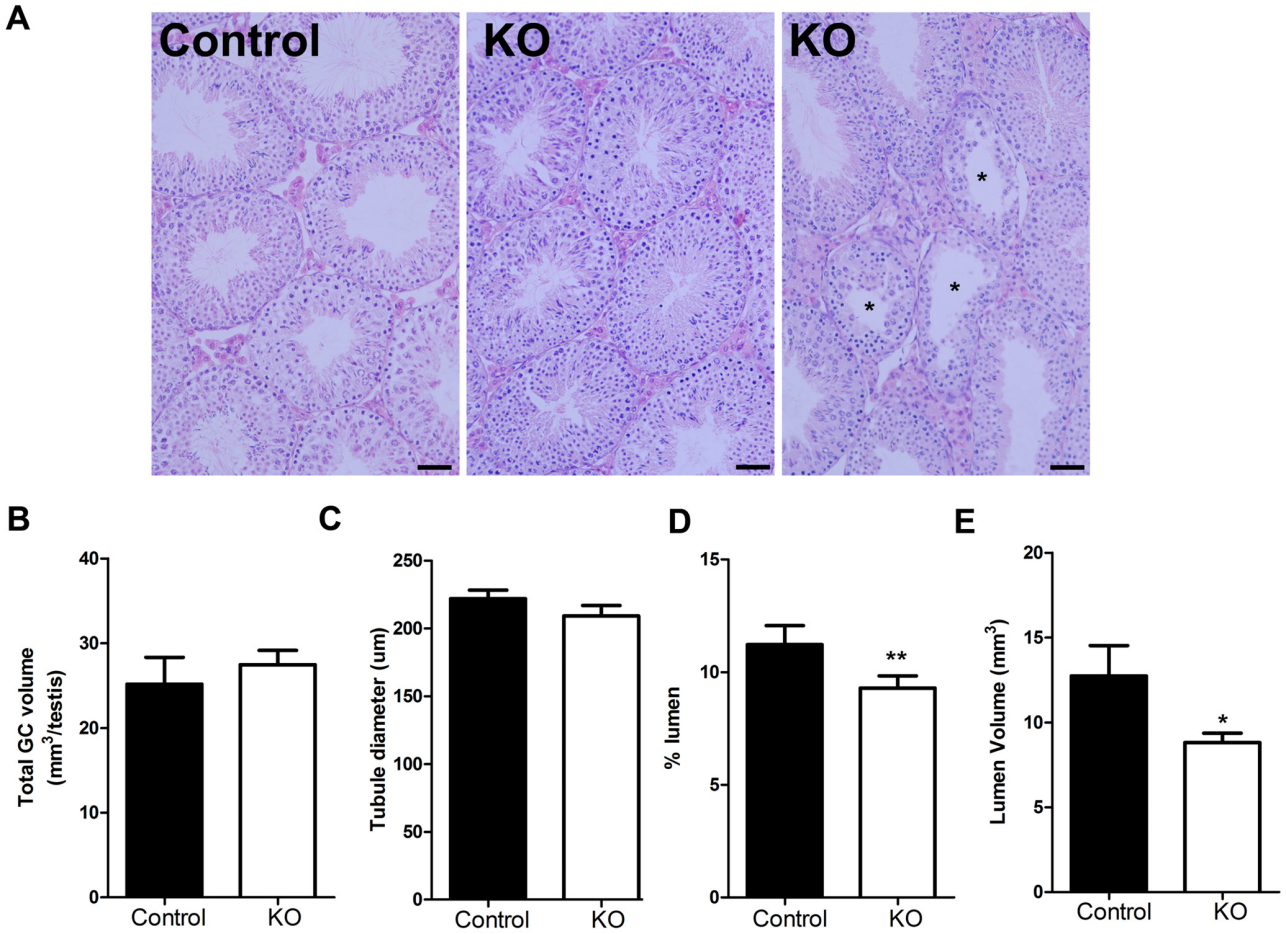
Histological comparison of adult SMARKO and control testes. **A.** Most seminiferous tubules in SMARKO testes looked comparable to control testes at d100, however, a small proportion (10%) were abnormal with disturbed spermatogenesis (*). Lumens appeared smaller in many seminiferous tubules in SMARKO testes at d100, compared to controls. **B.** There was no significant change in the total germ cell volume in SMARKO testes at d100, compared to controls. **C.** There was no significant change in seminiferous tubule diameter in SMARKO testes at d100, compared to controls. The percentage (**D**) **and volume** (**E**) of seminiferous tubule lumen was significantly reduced at d100 in SMARKO testes compared to controls. Scale bars  = 50 µm. Values are means ± S.E.M. (n = 4–6 mice), * p<0.05, ** p<0.01 compared to controls littermates.

Spermatogenesis and testicular histology appeared normal in the majority of seminiferous tubules from SMARKO males at d100 (Figure 3A); however, around 10% of seminiferous tubules appeared abnormal in SMARKO testes, with vacuoles and reduced depth of seminiferous epithelium and disrupted spermatogenesis (Figure 3A, right panel). This did not result in any significant change in seminiferous tubule diameter but there was a significant reduction in seminiferous tubule lumen volume per testis in SMARKOs at d100 (Figure 3C–E). There was no significant change in the total volume of GCs (Figure 3B) or in the number of any type of GC (data not shown) in adult SMARKOs, compared to control littermates.

### Impact of SMARKO on Leydig cell function and hormone profiles

**Figure 4:**
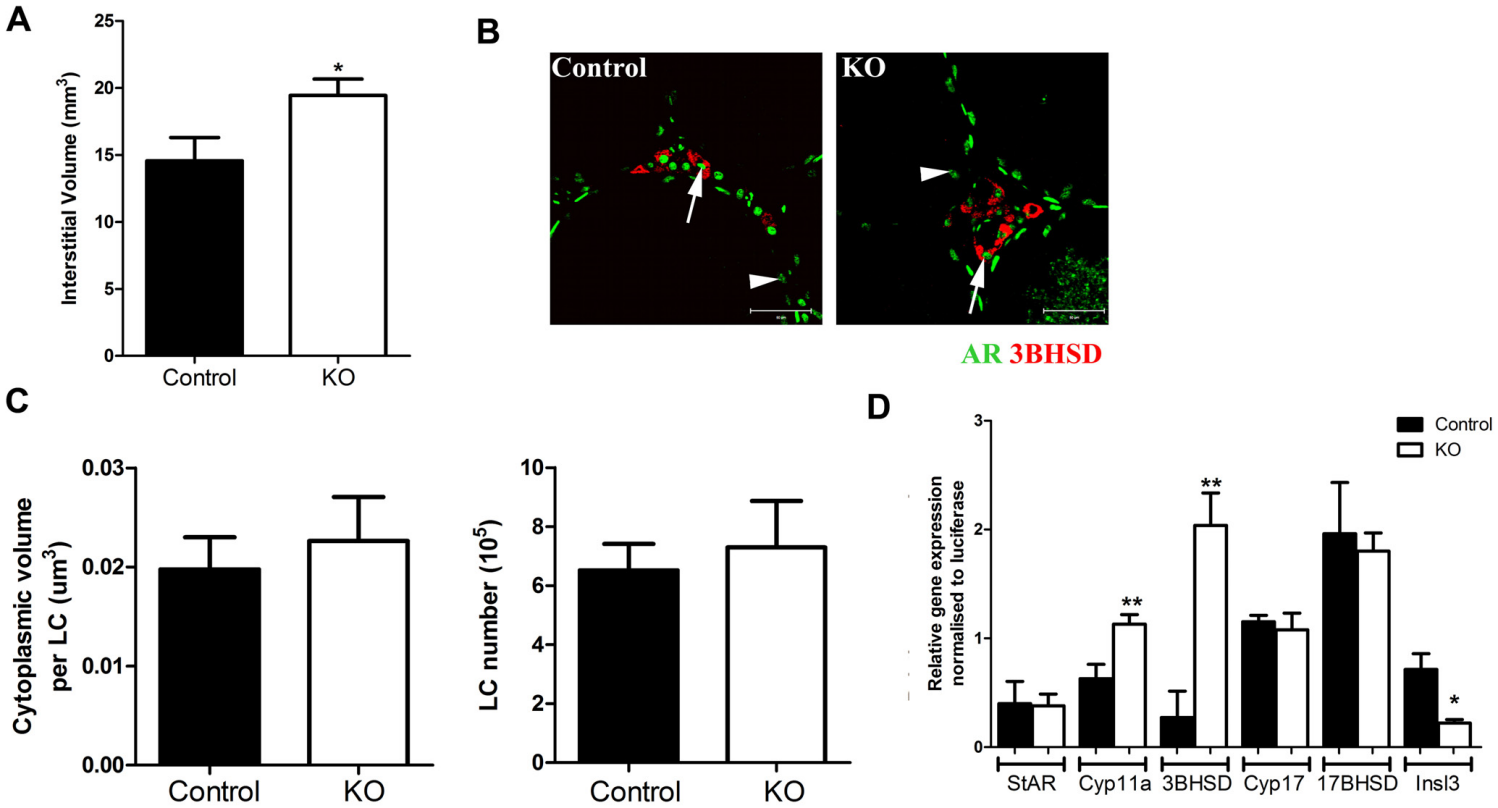
Evaluation of Leydig cell (LC) function in SMARKO testes. **A.** There was a significant increase in interstitial volume in SMARKO adult testes. **B.** Immunostaining for 3βHSD (red) and AR (green) demonstrating that LCs (arrow) and SCs (arrowhead) express AR in both SMARKO and control testes. **C.** Quantification of LC size and number highlighting no significant change in either in SMARKO testes, compared to controls. **D.** Relative expression of steroidogenesis enzymes (StAR, cyp11a, 3βHSD, Cyp17 and 17βHSD) and Inls3 in d100 control and SMARKO testes. Values are mean ± SEM (n = 4–6 mice), ** p<0.01, compared to controls littermates. Scale bars  = 50 µm.

**Figure 5:**
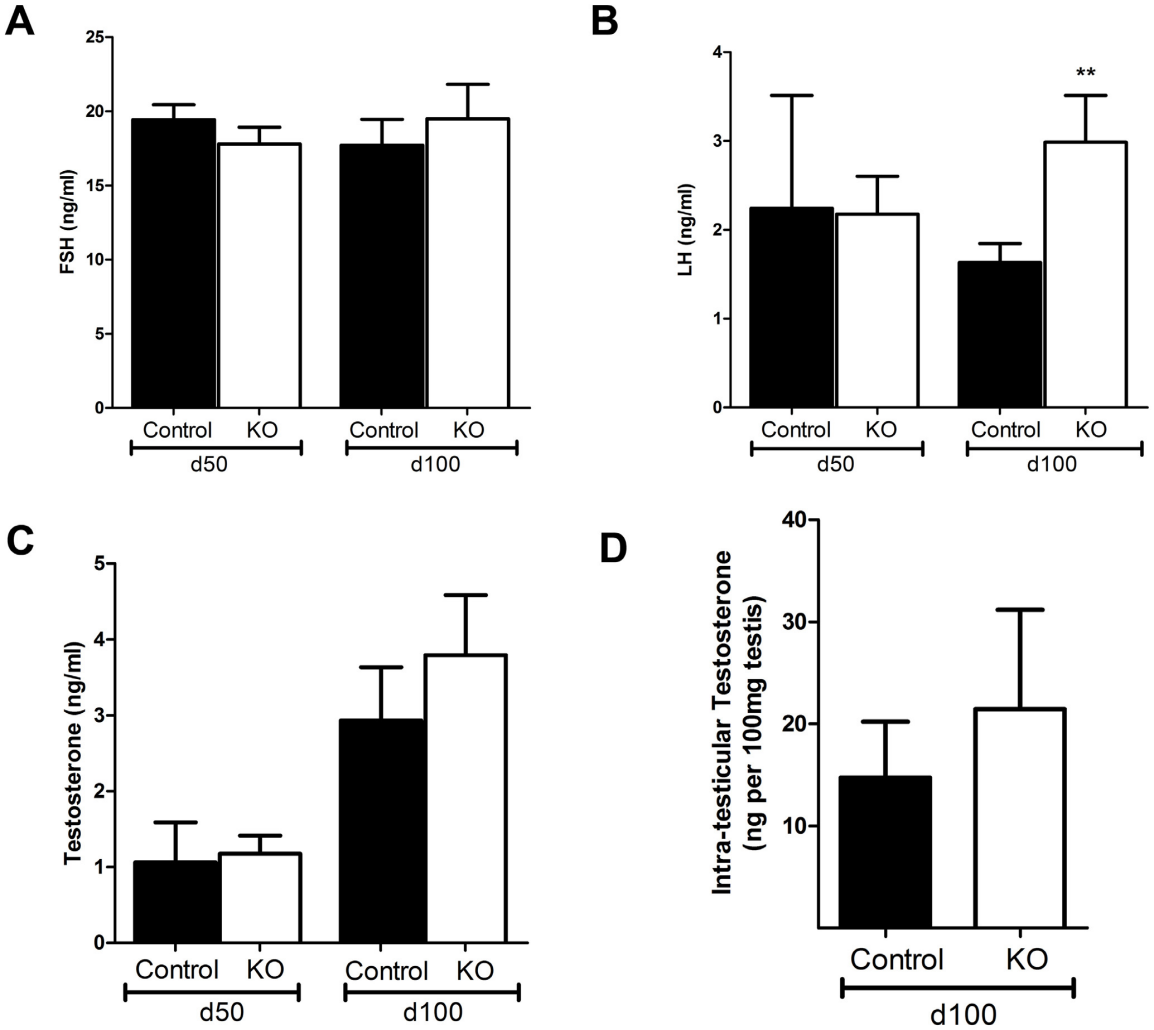
Hormone concentrations in SMARKO and control adult males. **A.** Serum follicle stimulating hormone (FSH) concentrations were not significantly different in SMARKO males, compared to controls, at d50 or d100. **B.** Serum luteinising hormone (LH) concentrations were significantly increased in SMARKO males at d100, but not at d50 compared to controls. **C.** Serum testosterone concentrations were not significantly different in SMARKO males, compared to controls at d50 or d100. **D.** Intra-testicular testosterone concentrations were not significantly altered in SMARKO males at d100. Values are mean ± SEM (n = 8–27 mice), ** p<0.01, compared to control littermates.

A significant increase in interstitial volume (Figure 4A), and percentage interstitium (data not shown) was observed in d100 SMARKOs, compared to control littermates. Testes were immunostained for 3β-HSD and AR to highlight that AR is expressed in LCs in both control and KO testes at d100 (arrow, Figure 4B). Quantification revealed that there was no significant increase in LC size and number in SMARKO mice at d100, compared to controls (Figure 4C). There was a significant increase in relative expression of cyp11a, 3βHSD, and Insl3 in SMARKO d100 testes compared to controls, while expression of other genes involved in steroidogenesis (namely StAR, Cyp17 and 17βHSD) were not significantly changed (Figure 4D). Serum follicle stimulating hormone (FSH) concentrations were not significantly changed at d100 in SMARKO males, compared to controls (Figure 5A). In contrast, serum Luteinizing hormone (LH) was increased in SMARKO males at d100, but not at d50, compared to controls (Figure 5B). Serum testosterone concentrations were not significantly altered in SMARKO mice at d50 or d100 compared to controls (Figure 5C) and there was no significant change in intra-testicular testosterone concentrations (expressed per 100 mg testis) in SMARKO testes, compared to controls (Figure 5D).

### Impact of SMARKO on SC function

**Figure 6:**
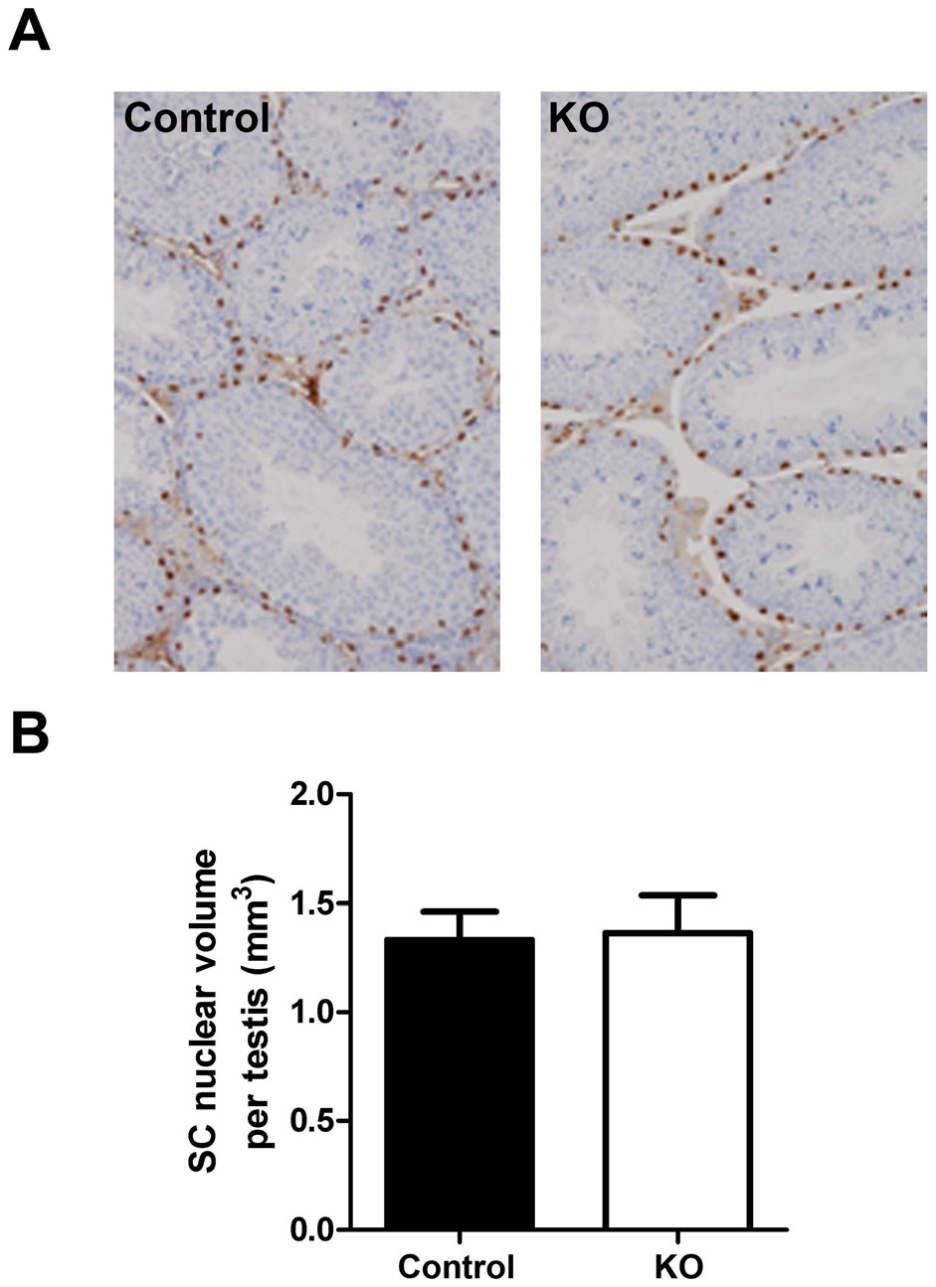
Evaluation of Sertoli cell (SC) function in SMARKO testes. **A.** WT-1 staining (black) was used to identify SC nuclei and highlighted their basal location in SMARKO and control males. **B.** SC mean nuclear volume in SMARKO and control d100 testes. Values are means ± S.E.M. (n = 4–6 mice). Scale bars  = 50 µm.

As expected, SC nuclei (identified by WT-1 expression) were observed at the base of seminiferous tubules in SMARKO and control mice at d100 and SC nuclear volume per testis (equates to SC number) was not significantly altered in SMARKO testes at d100, compared to controls (Figure 6). Furthermore, expression of SC-specific genes previously shown to be androgen-independent (androgen binding protein, ABP, [37]) or androgen-dependent (Rhox5, Eppin and Tubb3 [38]) were not significantly changed in SMARKO testes at d100, compared to control littermates (data not shown). Note that AR was expressed in SCs in both control and SMARKO testes at d100 (arrowhead, Figure 4B).

### Vasomotion in SMARKO testes

**Figure 7:**
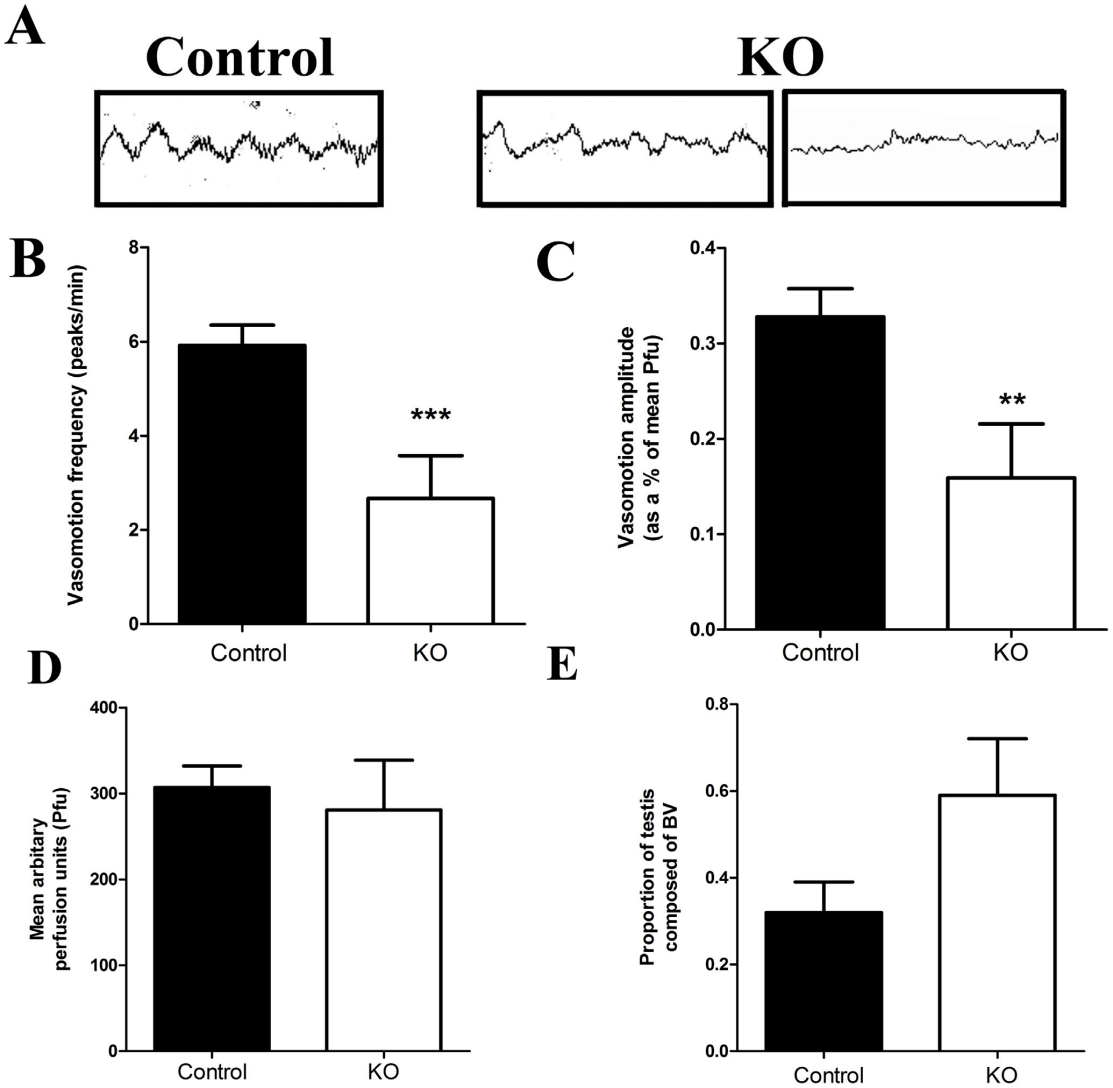
Vasomotion in SMARKO testes. **A.** Representative laser Doppler recordings of testicular blood flow in one control and two KO testes (1 minute each). **B.** Average vasomotion frequency was significantly reduced in d100 KO testes compared to controls. **C.** Average vasomotion amplitude was significantly reduced in d100 KO testes compared to controls. **D.** There was no significant change in average blood flow (Pfu) through d100 SMARKO testes compared to controls. **E.** Average proportion of blood vessels in d100 control and KO testes. Values are mean ± SEM (n = 3–14 mice), * p<0.05, compared to control littermates.

A regular high amplitude (sinus curve-like) vasomotion pattern was present in control testes (14 of 14 males), as expected (Figure 7A, right panel). Conversely, vasomotion persisted in 71% (5 of 7 males) of SMARKO testes (Figure 7A, middle panel) but was markedly disturbed while no vasomotion was detected in 2 SMARKO males (Figure 7A, right panel). Compared to controls, vasomotion frequency and amplitude were both significantly reduced in those SMARKO testes which retained some degree of vasomotion (Figure 7B and C respectively). There was no significant difference in average blood flow in SMARKO testes compared to controls (Figure 7D). There was a significant increase in the proportion of blood vessels in SMARKO adult testes compared to controls (Figure 7E); this appeared to be due to an increase in average blood vessel size (data not shown, p = 0.09) rather than any changes in the number of blood vessel cross sections per testis (data not shown, p = 0.8). There was no significant effect on VEGF-A mRNA expression in SMARKO testes at d21 or d100, compared to controls (data not shown).

### Response to hCG exposure

**Figure 8:**
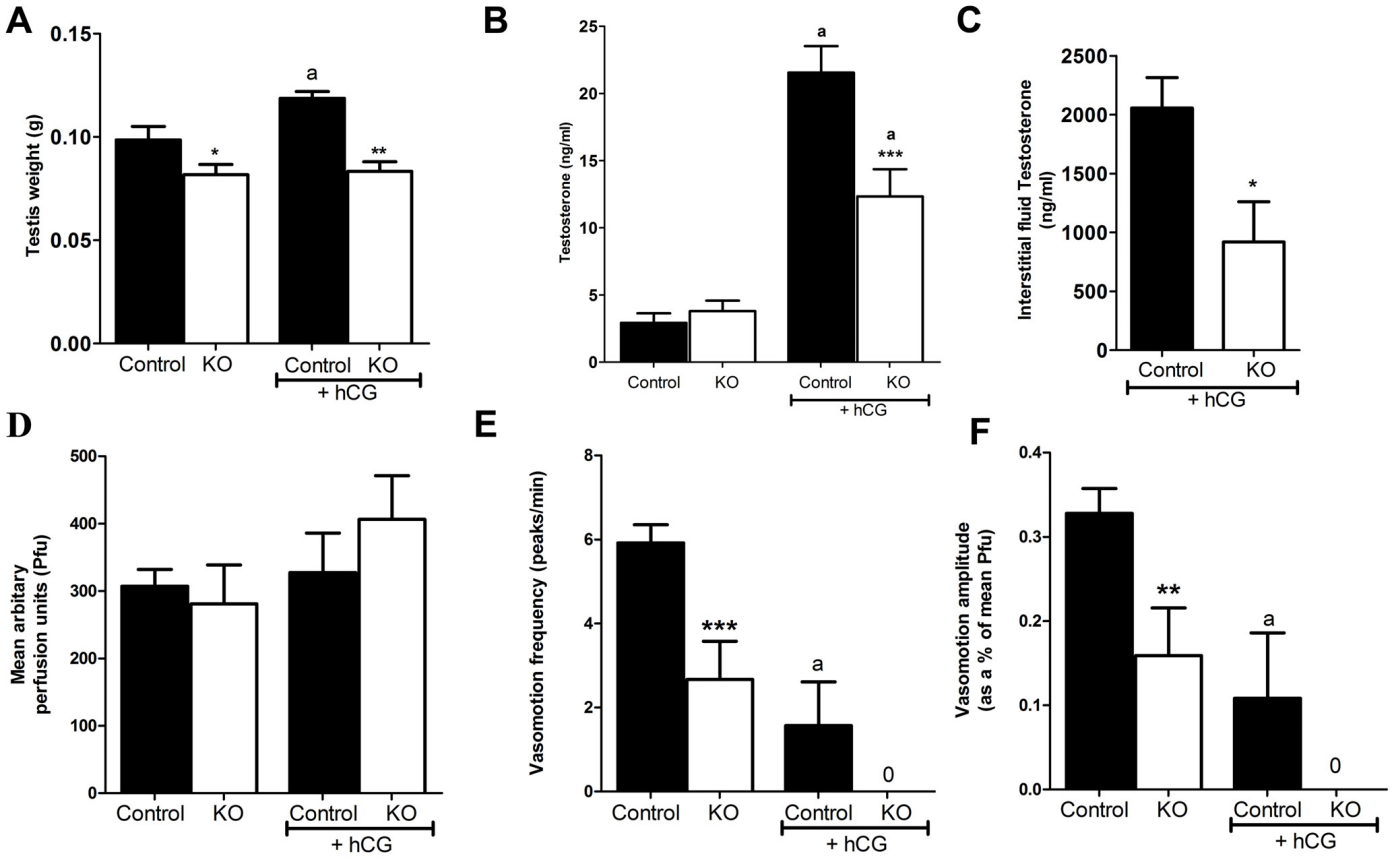
Testicular response to hCG exposure in SMARKO mice. **A.** Testis weight increased significantly in SMAR control, but not in SMARKO 16 hours after hCG treatment, presumably reflecting alterations in interstitial fluid volume. **B.** Serum testosterone concentrations increased significantly in both SMARKO and control adult males after exposure to hCG, but this increase was larger in controls than in KOs. **C.** Interstitial fluid testosterone concentrations were significantly higher in control testes than in SMARKOs at d100 after exposure to hCG. **D.** Average blood flow (Pfu) through adult SMARKO and control testes 16 hours after exposure to hCG. **E.** Average vasomotion frequency in adult SMARKO and control testes 16 hours after exposure to hCG. **F.** Average vasomotion amplitude in adult SMARKO and control testes 16 hours after exposure to hCG. Note that vasomotion frequency and amplitude were only measured in the two hcg-treated controls testes in which vasomotion could still be detected. Values are mean ± SEM (n = 5–7 mice), * p<0.05, ** p<0.01, *** p<0.001, compared to control littermates. ^a^ p<0.01, compared to untreated mice.

It is well documented that exposure to hCG increases blood vessel permeability and inhibits vasomotion resulting in increased IF volume in adult rats [20], [39], [40], [41]. In this study, exposure to hCG 16 h prior to cull resulted in a significant increase in testis weight in adult control mice, reflecting increased IF volume, but not in SMARKO adults (Figure 8A). hCG also significantly increased serum testosterone concentrations in both SMARKO and controls (Figure 8B), however, there was only a 3-fold increase in SMARKO mice compared to a 7-fold increase in control mice. IF testosterone concentrations were significantly increased in control mice after hCG exposure, compared to SMARKOs (Figure 8C). Exposure to hCG prevented vasomotion in 71% of control adult mice (5 of 7 males) and prevented vasomotion in all SMARKO adult mice (5 of 5 males). This lack of vasomotion in hCG treated control or SMARKO mice resulted in a small but not significant increase in testicular blood flow (Figure 8D). Vasomotion frequency and amplitude were both significantly reduced in the two hCG-treated control mice in which vasomotion persisted (Figure 8E and F).

## Discussion

The role for androgens in vascular function has been recognised for several decades [42], [43] yet the cellular mechanisms remain rather elusive. For example, it is not clear how testosterone regulates testicular blood flow or what role testicular arteriole smooth muscle ARs play in testicular function. Mice completely lacking a functional AR have been used to study the effects of androgens on the cardiovascular system [44], [45], however, the effects on the testes cannot be studied in these mice as they have reduced circulating testosterone concentrations and cryptorchidism and AR is deleted from all cell types therefore potentially masking the effects of AR in the blood vessels. We therefore investigated the effects of androgens on the testicular vasculature directly using an arteriole smooth muscle cell specific ARKO mouse (termed SMARKO) [8]. We demonstrate that ablating arteriole smooth muscle AR has no gross effect on spermatogenesis or on male fertility. However, detailed analysis revealed that LC function, vasomotion and fluid dynamics within the testis were impaired in SMARKO mice, indicating a role for androgen signalling via the blood vessels in locally regulating LC function and microvascular blood flow.

Ablation of AR from the vascular smooth muscle cells had no effect on reproductive tract formation, testes descent or adult prostate or seminal vesicle size. Conversely, testis size was significantly reduced in adulthood, but was not affected prior to puberty (i.e. at d12, 21 or 35). Note though that testis size does not significantly change between d100 and d300 and the degree of histological abnormalities was not increased in d300 SMARKO testes compared to d100 testes (data not shown), suggesting that lack of AR in the smooth muscle cells of the blood vessels causes an adult testicular phenotype but that this phenotype does not deteriorate with aging. Reduced adult testis size most commonly reflects reduced GC number per testis, but this was not the case in SMARKO males which were normally fertile and exhibited only focal areas of deficient spermatogenesis. This is in contrast to PTM-ARKO and SCARKO mice which are azoospermic, have a more homogeneous testicular phenotype and are infertile [7], [8]. The patchy loss of GCs in SMARKO testes could reflect focal hypoxia, which has been shown to result in GC death triggered by DNA damage [46]. The testis normally maintains a low oxygen tension [47], therefore any significant decrease in testicular blood flow would likely result in hypoxia and testicular damage. Overall blood flow was normal in SMARKO testes and only focal areas of atrophy were identified as discussed above. It is possible that ablating AR from the smooth muscle cells of the arterioles only disturbs testicular microcirculation at its most distal parts, resulting in focal areas of azoospermia in these regions. More general hypoxic effects may be limited by the observed ‘compensatory’ increase in blood vessel size and overall blood vessel volume. Interestingly, there was no significant difference in the proportion of blood vessels in SMARKO testes at d21 (data not shown) suggesting that any increase in blood vessel size in these mice must happen after puberty, the age at which vasomotion begins [20]. Therefore, altered vasomotion could be one of the triggers for increased blood vessel size in these mice; this requires further investigation.

As GC number was not significantly reduced in SMARKO testes, the reduction in testis size in these animals is most probably due to the reduction in seminiferous tubule lumen volume. Lumen volume reflects seminiferous tubule fluid (STF) production by SCs, which is androgen-dependent [7], [48], [49]. However, serum and intra-testicular testosterone concentrations were normal in adult SMARKO males, SCs normally expressed AR, and the expression of androgen-dependent SC genes such as Eppin or Rhox 5 [7], [8] were also unchanged. Therefore the deficits in STF production in SMARKOs are not explained by changes in androgen action on SCs; further investigations are required to identify the mechanisms underlying the apparent reduction in STF volume in these mice. Some insight could be gained from observations in other testicular compartments in SMARKO mice. For example, the increased relative expression of 3βHSD and cyp11a in SMARKO d100 testes compared to controls is indicative of a compensatory increase in some of the steroidogenesis enzymes in order to maintain normal serum testosterone concentrations. Furthermore, serum LH concentrations were significantly elevated in SMARKO adult males while serum and intra-testicular testosterone concentrations were unaltered; this is in line with the concept of compensatory LC failure and is in agreement with the phenotype reported by Zhang et al when using the same SM22-Cre to ablate AR expression [35]. Interestingly, as seen with SCs, LCs from SMARKO mice appeared normal for most aspects investigated such as number, size and AR expression but had reduced mRNA expression of the LC differentiation marker Insl3. These findings therefore suggest that signalling via arteriole smooth muscle AR affects LC function and differentiation, but our results do not allow us to distinguish whether ablation of AR from arteriole smooth muscle cells directly affects the LCs by unknown signalling mechanisms or whether this impaired LC function and differentiation is the result of altered fluid transport to and from the testis, or both. Transport of fluids into the testis is perhaps more likely to be affected than transport out of the testis, as AR is deleted from the testicular arterioles of SMARKO males, which are responsible for delivery of blood to the testis, rather than removal which occurs via venules. Conversely, if testosterone was getting trapped in the testes due to impaired transport out of the testis, intra-testicular testosterone would be increased and this was not seen in SMARKO mice. The hypothesis of impaired fluid delivery into the testis could also offer some explanation for the reduction in STF production as delivery of FSH, which can affect STF production in immature rats [49], may also be impaired in SMARKO mice. However, relative expression of ABP, an FSH-dependent SC gene [50], is not significantly altered in SMARKO adults compared to controls suggesting that FSH delivery to and action in the testis is normal. Furthermore, it has been shown that LH can affect vascular permeability, and so fluid exchange, by increasing VEGF production [12], [18], [19], but testicular VEGF mRNA expression was not significantly changed in adult SMARKOs, despite elevated LH concentrations. This suggests that VEGF-mediated changes in vascular permeability cannot explain the phenotype observed in SMARKO testes. Our results therefore suggest that testicular arteriole smooth muscle AR plays a role in regulating normal LC function, possibly by mediating testicular fluid exchange and microvascular blood flow within the testis but the mechanisms need further investigation.

Fluid transfer between blood vessels and IF is mediated by vasomotion in the testes [20], [21], [22]. When blood is flowing quickly through the microvasculature, plasma filters from the blood into the interstitium and vice versa when blood flows slowly [22]. As discussed above, there was no significant change in overall blood flow in SMARKO testes, compared to controls, but vasomotion was reduced in SMARKO testes. Previous publications have shown that testosterone is required for vasomotion [17], [26] but it is not known how or via which cells this effect is mediated. Our results demonstrate for the first time that arteriole smooth muscle AR is necessary for normal testicular vasomotion. However, it unclear if this impaired vasomotion in SMARKO testes is due to a lack of arteriole smooth muscle AR itself or if it is a secondary problem due the testicular abnormalities such as the compensatory LC failure. Reduced vasomotion but normal blood flow in SMARKO testes suggests that, as opposed to the cyclical fast and slow microvascular blood flow normally observed in the testes, blood is likely to be constantly flowing quickly through SMARKO testes which would reduce resorption back into the bloodstream and result in an increase in IF volume. IF volume was not measured directly in the present studies due to technical limitations, but it is likely to be increased in SMARKO adult testes as, when quantified stereologically, interstitial volume was increased without any changes in LC volume. This pattern of continuous blood flow through SMARKO testes is similar to that previously shown to occur in control rats treated with hCG which impairs vasomotion [20], [39], [40], [41]. Serum LH, which signals through the same receptors as hCG, was increased in SMARKO males which could also offer some explanation for the impaired vasomotion observed in these mice.

Previous researchers have shown that hCG exposure abolishes vasomotion and increases blood vessel permeability in control rats thereby increasing IF volume [39], [40], [41] but, to the best of our knowledge, it is not known if mice respond in the same way; we therefore tested this in our mouse model. Little is known about how hCG (or elevated LH) affects vasomotion but it is unlikely to act simply by increasing testosterone as supra-physiological concentrations of testosterone do not prevent vasomotion in rats [22]. As expected, hCG induced a significant increase in serum testosterone in both control and SMARKO mice suggesting that hCG is being delivered to the testis, that the LCs are able to respond by producing more testosterone and that this testosterone is able to leave the testis and enter the bloodstream. However, the increase in serum testosterone was greater in the hCG treated controls than in the KOs, providing further evidence that LCs are not able to respond to stimulation in SMARKOs as well as in controls. This may be due to the LC compensatory failure observed in SMARKO testes which could mean that LCs are already working close to their maximal level so increased stimulation by hCG would not induce as great a LC response as that seen in controls. This link between arteriole smooth muscle AR signalling and LC function requires further investigation.

As expected, testis size increased in all hCG-treated controls, presumably due to an increase in IF, and vasomotion was absent in most control testes 16 hours after hCG exposure. It is not clear why vasomotion was still present in 2 control mice treated with hCG, however both amplitude and frequency of vasomotion was significantly reduced in these mice, compared to untreated control mice. It is possible that the dose of or timing of hCG is not optimal to inhibit vasomotion in all mice or that mice may respond differently to hCG than rats do; this warrants further investigation. Interestingly, unlike control testes, SMARKO testes did not increase in size 16 hours after hCG treatment but vasomotion was completely abolished in all SMARKO males. The lack of testicular size increase in SMARKO males could be a direct result of ablating smooth muscle AR but is more likely to reflect the already increased IF volume in untreated SMARKO testes or the impaired blood flow and/or LC function in SMARKO males.

Together these results demonstrate that androgen action via smooth muscle cells of testicular arterioles is important in regulating LC function, vasomotion and testicular fluid dynamics but is not essential for normal testis development or fertility. However, it is not clear whether arteriole smooth muscle AR signalling directly affects LC function which then affects blood vessel function, or vice versa, or both. Further investigations are required to understand the underlying mechanisms.
